# Left-Handed Cardiac Surgery Learning Lab: An EACTS Annual Meeting Initiative

**DOI:** 10.1093/icvts/ivaf279

**Published:** 2025-11-26

**Authors:** Eric E Vinck, Mona Bickel-Dabadghao, Alicja Zientara, Anna Lena Emrich, Nora Göbel, Sabine Bleiziffer, Peyman Sardari Nia, Roman Gottardi

**Affiliations:** Department of Cardiothoracic Surgery, Maastricht University Medical Center, Maastricht University, Maastricht, 6229 HX, The Netherlands; Department of Cardiac Surgery, International Hospital of Colombia, Cardiovascular Institute, Floridablanca, 81001, Colombia; Department of Cardiac Surgery, Central Hospital Bad Berka, Bad Berka, 99438, Germany; Department of Cardiovascular Surgery, University Heart Center Freiburg–Bad Krozingen, Freiburg, 79106, Germany; Department of Cardiovascular Surgery, University Medical Center Mainz, Mainz, 55131, Germany; Department of Cardiovascular Surgery, Robert Bosch Hospital, 70376, Stuttgart, Germany; Department of Cardiac Surgery, Central Hospital Bad Berka, Bad Berka, 99438, Germany; Department of Cardiothoracic Surgery, Maastricht University Medical Center, Maastricht University, Maastricht, 6229 HX, The Netherlands; Department of Cardiovascular Surgery, University Heart Center Freiburg–Bad Krozingen, Freiburg, 79106, Germany; Department of Cardiothoracic and Vascular Surgery, Westpfalz Klinikum, 67655, Kaiserslautern, Germany

**Keywords:** left-handed, cardiac surgery, learning lab, EACTS

## Abstract

Left-handed cardiac surgery has been a rising topic. Training necessities pertaining to left-handed cardiac surgery education and mentorship are crucial to this group of surgeons. Through initiatives of experienced left-handed cardiac surgeons in association with the EACTS, a left-handed learning lab at the EACTS annual meeting was developed. This skills lab training is dedicated to offering left-handed surgeons a space which offers both technical development and career networking. In this short communication, we detail lessons learned and future insights into left-handed cardiac surgery education through hands-on training.

## INTRODUCTION

Medical and surgical societies play a crucial role in fostering professional development through annual scientific meetings. These meetings encourage networking, education, and hands-on training by offering both dry and wet labs. In a right-hand-dominated specialty, only about 5% of cardiac surgeons operate exclusively with their left hand.^1–3^ Consequently, few educational resources and training tools exist to support left-handed (LH) cardiac surgeons. A recent survey found that 78.1% of cardiac surgery residents had no exposure to LH mentorship during training.^1–3^ Furthermore, 63.5% reported being pressured to operate right-handed, and 47.3% expressed a need for LH faculty guidance.^1–3^ Among LH surgeons, 52.8% are left-handed operators, 73% remain true left-hand operators, while 18.1% converted to right-handed surgery due to external pressure.^1–3^ In response to a growing demand for mentorship and training tailored to left-handed surgeons, a wet lab designed exclusively for left-handers was created. At the 2024 European Association for Cardio-Thoracic Surgery (EACTS) Annual Meeting in Lisbon, Portugal, the first pilot left-handed cardiac surgery learning lab was introduced led by experienced faculty. Following notoriously positive feedback from this inaugural event, a second edition of the LH learning lab was held at the 2025 EACTS Annual Meeting in Copenhagen, Denmark. This report summarizes our experience.

## THE LEFT-HANDED LEARNING LAB INITIATIVE

**Figure 1.**
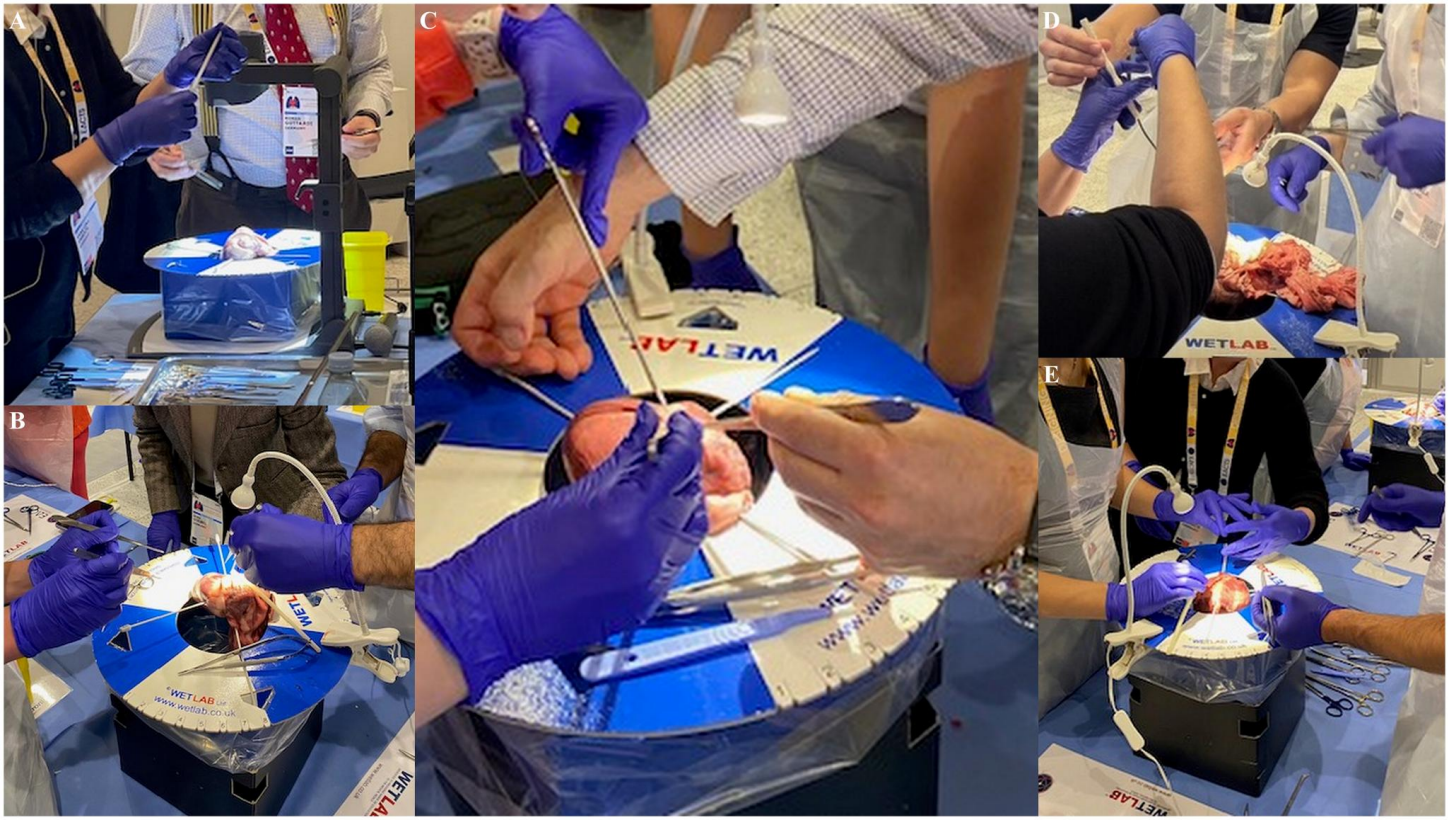
(A) Opening Demonstration of CABG Anastomosis Using the Left Hand. (B and C) Personalized Guidance from Left-Handed Surgeons. (D and E) Left-Handed Instructors at Different Training Stations Guiding Left-Handed Participants

At the first session in 2024, participants mainly practiced coronary anastomosis in addition to some aortic valve replacements while instructors assessed their technical needs and skill levels. Nine participants attended this initial session. The importance of such a learning environment is that it allows LH surgeons to discuss challenges freely, share strategies, and practice in a supportive, nonjudgmental setting. Participants also identified additional surgical areas they hoped to explore in future labs. Building on these insights, the 2025 Copenhagen session was refined and improved. Participants in the 2025 session represented three distinct groups: (1) students and residents early in their careers without LH faculty support; (2) left-handed surgeons who had been trained or compelled to operate right-handed and wished to reattempt procedures using their dominant hand; (3) curious LH surgeons seeking community and technical exchange. This diversity created a rich learning environment with varied expectations and experiences. The session began with a live demonstration of a distal coronary anastomosis performed by a LH surgeon (**Figure 1A**), sparking an open debate on “How do you do it left-handed?” The instructors themselves showcased diverse approaches—underscoring the adaptability required in LH surgical practice (**Figure 1B and C**). Participants worked in pairs under continuous guidance from experienced LH mentors. Core techniques included coronary artery anastomosis and aortic valve replacement. Faculty rotated among stations, offering personalized instructions while discussing technique variations (**Figure 1D and E**).

### Participant survey results

Of the 9 participants attending the 2025 LH Learning Lab, 8 consented to publish their feedback anonymously through an electronic survey sent to the participants finalizing the learning lab. Their backgrounds included 2 pre-trainees, 4 residents, and 2 attending surgeons. All participants used their left hand predominantly for 50%-75% of procedures. Half reported always being permitted to operate left-handed during training, 37.5% were sometimes allowed, and 12.5% were prohibited from doing so. Only 25% had access to an LH mentor. Despite these challenges, 87.5% reported gaining substantial takeaways from the learning lab, and all participants intended to apply new insights directly to their practice. 62.5% felt more confident operating after the lab, 25% somewhat more confident, and 12.5% unchanged (an experienced LH surgeon). Overall satisfaction was unanimous; the lab filled a significant training gap and received a 5/5-star rating.

## WHAT WE LEARNED SO FAR

**Figure 2.**
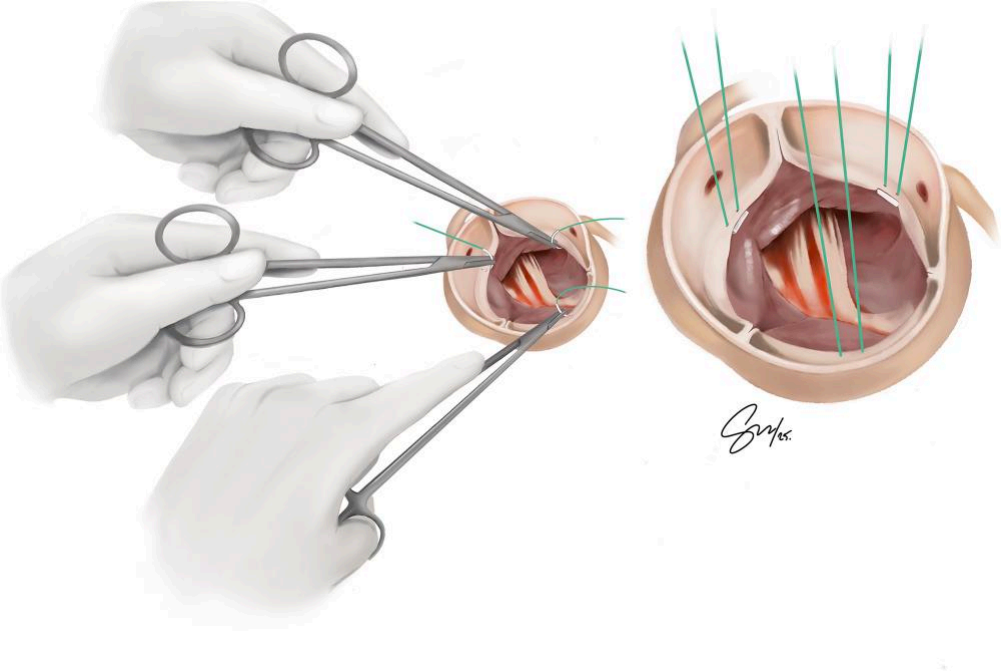
Sub-Annular Aortic Valve Suture Placement Performed by a Left-Handed Surgeon

**Figure 3.**
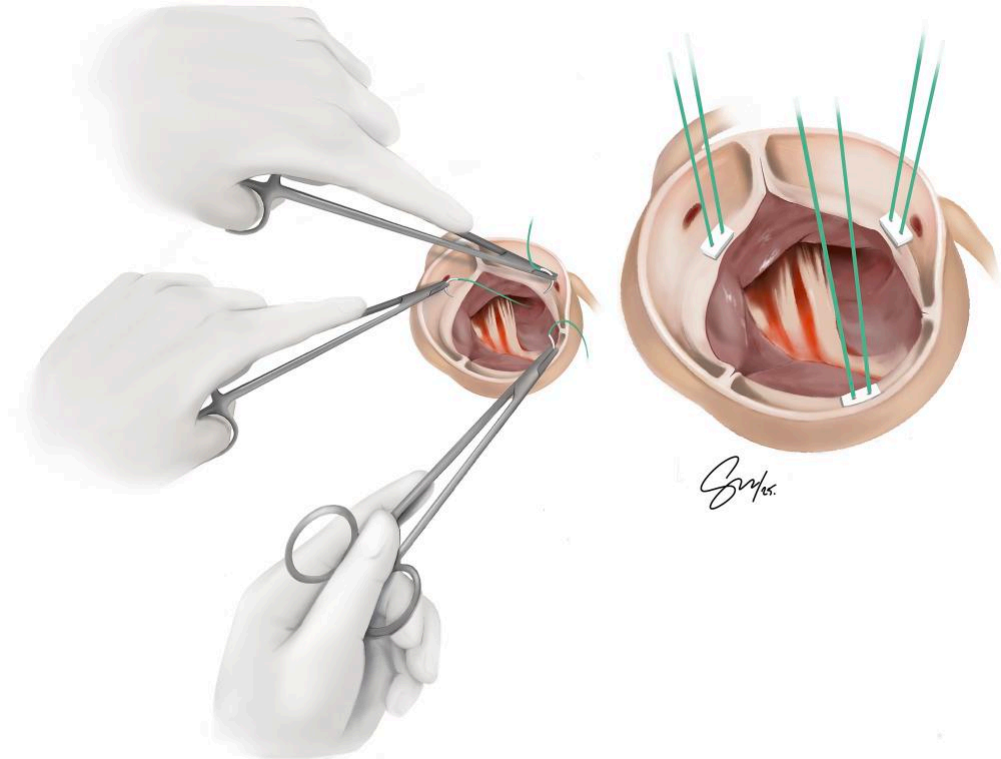
Supra-Annular Aortic Valve Suture Placement Performed by a Left-Handed Surgeon

In cardiac surgery, training is not always standardized, and for LH surgeons, this lack of structure and learning resources is even more pronounced. As a result of this scarcity of mentorship and training tools, technical proficiency among LH surgeons is highly individualized. Many left-handers are compelled to find creative and often isolated solutions, with some pressured to “just use the right hand.”^1–3^ LH mentorship should begin early in training, with right-handed instructors encouraged to support LH residents rather than discouraging their natural dominance.^1–3^ While any skilled surgeon can offer mentorship, only an experienced LH operator can teach left-handed nuanced ergonomics, adaptations and tricks. Even between experienced LH cardiac surgeons, left-handed technical approaches vary greatly; these variations offer a broader range of surgical options and an expanded armamentarium that trainees can learn and benefit from once they have access to LH mentors. **Figures 2** and **3** illustrate both sub-annular and supra-annular approaches for aortic valve suture placements performed by a left-handed surgeon. In this variation, the non-coronary sinus valve sutures are placed using a Left-forehand technique for a sub-annular approach and a Left-backhand technique for a supra-annular approach. For sub-annular aortic valve sutures, both the left and right coronary sinus sutures are placed using a left-backhand approach (**Figure 2**). For supra-annular aortic valve sutures, both the left and right coronary sinus sutures are placed using a left-forehand technique (**Figure 3**).

During the wet lab, left-handed instruments were provided. We discovered that left-handed surgeons experienced with right-handed instruments found LH tools unfamiliar and uncomfortable. Whereas trainees with limited prior exposure to right-handed instruments adapted quickly and felt more natural using the same-hand-dominant tools. This raises the question: are LH instruments essential, or simply advantageous? Also, should left-handed instruments be introduced at a late stage or not? Since LH instruments are costly and rarely produced, their acquisition poses financial challenges. Instead, we recommend trainees without access to LH tools to master right-handed instruments without delaying surgical progress. Most importantly, LH trainees must be allowed to operate using their dominant hand.

The camaraderie and validation participants experienced when interacting with other LH surgeons at this learning lab were insightful and invaluable. Future goals include upcoming lab modules covering sternal opening and cannulation techniques, and expanding the LH Learning Lab into a structured mini-fellowship or surgical rotation. Key takeaways expressed by the participants included: adapting body positions to optimize LH ergonomics, guiding assistants effectively, alternating between backhand and forehand sutures smoothly, exploring LH variations for valve and coronary procedures, networking, and using right-handed instruments efficiently with the left hand.

## CONCLUSIONS

Left-handed cardiac surgeons and trainees face unique learning challenges. Early mentorship from LH faculty accelerates both technical proficiency and professional development. The EACTS Left-handed Learning Lab provides an essential platform for LH surgeons to exchange knowledge, refine technique, and foster a supportive community—ultimately enhancing patient care.

## Data Availability

The data underlying this article will be shared on reasonable request to the corresponding author.
